# A Novel Cloud-Based Service Robotics Application to Data Center Environmental Monitoring

**DOI:** 10.3390/s16081255

**Published:** 2016-08-08

**Authors:** Ludovico Orlando Russo, Stefano Rosa, Marcello Maggiora, Basilio Bona

**Affiliations:** 1 Department of Control and Computer Engineering, Politecnico di Torino, Corso Duca Abruzzi 24, Turin 10129, Italy; stefano.rosa@polito.it (S.R.); basilio.bona@polito.it (B.B.); 2 Infrastructure IT Division, Politecnico di Torino, Corso Duca Abruzzi 24, Turin 10129, Italy; marcello.maggiora@polito.it

**Keywords:** cloud robotics, service robotics, environmental monitoring, data center, energy management

## Abstract

This work presents a robotic application aimed at performing environmental monitoring in data centers. Due to the high energy density managed in data centers, environmental monitoring is crucial for controlling air temperature and humidity throughout the whole environment, in order to improve power efficiency, avoid hardware failures and maximize the life cycle of IT devices. State of the art solutions for data center monitoring are nowadays based on environmental sensor networks, which continuously collect temperature and humidity data. These solutions are still expensive and do not scale well in large environments. This paper presents an alternative to environmental sensor networks that relies on autonomous mobile robots equipped with environmental sensors. The robots are controlled by a centralized cloud robotics platform that enables autonomous navigation and provides a remote client user interface for system management. From the user point of view, our solution simulates an environmental sensor network. The system can easily be reconfigured in order to adapt to management requirements and changes in the layout of the data center. For this reason, it is called the virtual sensor network. This paper discusses the implementation choices with regards to the particular requirements of the application and presents and discusses data collected during a long-term experiment in a real scenario.

## 1. Introduction

This work concerns the development and testing of an environmental monitoring system for data centers, which relies on autonomous mobile robots and is based on the cloud robotics paradigm. The system is able to monitor environmental physical quantities (temperature and relative humidity) and to safely interact with fixed obstacles, moving obstacles and people working in the area.

The envisioned scenario is of crucial interest: an automatic and precise mapping of the temperature and humidity distributions in such environments is fundamental to take informed action to increase power efficiency. The designed robotic system is provided with autonomous navigation capabilities to autonomously move within the environment and collect environmental data.

The system is functionally equivalent to an Environmental Sensor Network (ESN), as it is able to periodically capture and store localized environmental measurements. For this reason, in the reminder of the paper, we refer to it as a Virtual Sensor Network (VSN), and we call localized sources of measurements VSN nodes. Collected data are reported to the user via a web-based Graphical User Interface (GUI), accessible from a web browser. With respect to an ESN, our system has the advantage of being completely reconfigurable, since the user can simply add or remove VSN nodes from a remote interface.

The aim of this paper is two-fold: firstly, it aims at presenting and discussing data collected by the system in a real case scenario, in order to demonstrate that the system is a valid alternative to state of the art ESNs; secondly, it aims at summarizing the authors’ previous works on the problem of data center monitoring [1,2] in order to provide a comprehensive and self-consistent vision on the problem and the developed solution.

This paper is organized as follows: The rest of this Section introduces the problem of data center energy management and reports the current state of the art solutions to data center monitoring, robot navigation and cloud robotics. Section 2 introduces the proposed solution, focusing on the architecture of the cloud robotic platform and the functional architecture of the robot service. Section 3 focuses on the particular autonomous navigation choices dictated by the scenario. Section 4 deepens the service application. Section 5 illustrates experiments and discusses collected data. Finally, Section 6 draws conclusions.

### 1.1. Energy Management in Data Centers

A data center is a facility housing servers, networking and storage hardware. It incorporates redundant power, cooling, fire suppression and security systems, as well as network connectivity.

Management of data centers is very expensive, both in terms of reliability assurance and in terms of energy consumption. Reliability assurance is related to the costs for planning, deploying and managing the equipment in order to provide a near 100% uptime: this is mission critical, since hard failures will reduce productivity. Energy consumption is related to the fact that data centers consume huge amounts of energy.

An improvement of the energy efficiency will give important results both for the owners of the data centers and for the whole community, since it would be in accordance with the energy saving goals established by the European Council in the objective known as the 20-20-20 targets (20% increase in energy efficiency, 20% reduction of CO${}_{2}$ emissions and 20% in the use of renewable energy by 2020) [3]. In fact, data centers are the most intensive energy consumption buildings in the world [4,5,6]. For instance, [4] reports that, in 2011, the total electricity used by data centers worldwide was between the 1.1% and 1.5% of the total electricity demand.

The Power Usage Effectiveness (PUE), defined as:

(1) $$PUE=\frac{\text{Total}\;\text{Facility}\;\text{Input}\;\text{Power}}{\text{Total}\;\text{IT}\;\text{Equipment}\;\text{Power}}$$

is a metric to measure the power efficiency of a data center. The average PUE rating in 2015 was 1.7 [6], meaning that there is a 70% power consumption overhead in the average data center compared to the power needed by the equipment.

Advances in microprocessor technology lead to both miniaturization of electronic components and increase in clock rates, increasing the computing power of a single chip. This increases the heat production in the rooms that must be expelled, since high temperature is the main cause of electronic hardware failures [7]. Hence, the main overhead of data center consumption happens in the cooling equipment [8].

**Figure 1:**
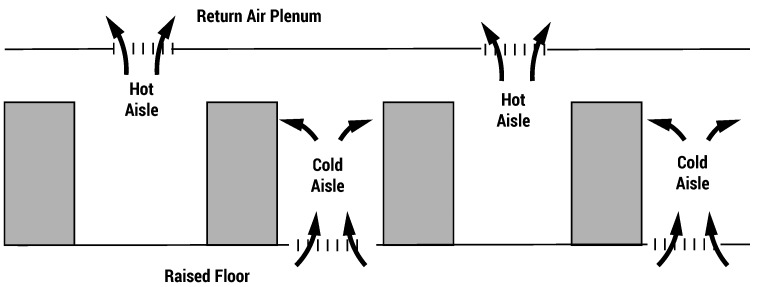
Typical data center hot/cold aisles layout.

The most common cooling solutions are the air cooled systems that distribute air with proper cooling capacity in the server rooms. The so-called hot/cold aisle layout (see Figure 1) maximizes the air cooling capacity. It consists of organizing racks in alternating rows with cold air intakes facing one way and hot air exhausts facing the other. In this way, alternate hot and cold aisles are created between the rows of racks. This layout avoids cold air mixing with hot exhausted air, improving efficiency, but is still affected by some critical issues, represented by the so-called hot spots.

Hot spots are local areas of excess temperature caused by a local lack of cooling capacity or inability to deliver the cooling where it is needed. They cause either an over-cooling of the environment (for compensating a localized problem) or the damage of electronic equipments (if not detected). According to [9], 84% of organizations experienced issues with data center power, space and cooling capacity, assets or uptime in the past few years. Various strategies to deal with hot spots can be adopted. These strategies are based on a thorough evaluation and assessment of the data center (e.g., balancing the thermal load or changing the air distribution planning). However, identification of hot spots still remains a problem only partially solved.

Data center monitoring groups all of the solutions aimed at measuring environmental quantities in server rooms for diagnostic and controlling purposes. It is devoted to reducing the cooling and management costs and the risks of hardware failures. Critical quantities are air temperature and relative humidity, which must be maintained within the ranges 20–24 C and 40–55 % to maximize the hardware life cycle [10].

Classical approaches to data center monitoring rely on localized cooling and a distributed metrology layer [11,12,13,14,15,16,17]. However, the dimensions of a typical data center room make it very difficult to instrument the whole environment with a dense ESN. In fact, in practical applications, managers adopt sensors to monitor only the perimeter of the room, while other parts are periodically inspected by human operators. This increases costs and risks.

Recently, mobile robots have been adopted to solve this problem: [18] proposes a multi-robot system where robots communicate with each other over a wireless network. Localization is based on Near-Field Communication (NFC) tags placed on the floor. However, this solution requires physical intervention on the environment and does not adapt to changes in layout. Moreover, it does not provide obstacle avoidance to allow the robots to safely move when people are operating in the room. A similar solution is proposed in [19], where a robot takes advantage of the standard square tiles on the data center floor to navigate. The approach guarantees complete coverage, but lacks autonomy and robustness.

### 1.2. Mobile and Cloud Robotics: An Overview

In recent years, autonomous robots and automated systems have been increasingly used in the industrial field in general [20,21,22] and in environmental monitoring in particular [23,24]. With respect to Automated Guided Vehicles (AGV) [25,26], mobile robots are able to navigate in non-instrumented environments, since they rely only on onboard sensors to perform autonomous navigation.

Autonomous navigation groups all of the techniques and algorithms that are necessary for a mobile robotic platform in order to accomplish three fundamental tasks: self-localization inside a known environment; building a map of an unknown environment; obstacle avoidance and path planning [27].

Localization is the problem for the robot to estimate its location relative to the environment. Correct and reliable localization with respect to a known map is one of the most fundamental problems in mobile robotics. Extended Kalman Filters (EKF) and Monte Carlo Localization (MCL) methods are the most common filtering algorithms used for robot localization [27].

The problem of map estimation in an unknown environment has been treated extensively in robotics, and the corresponding framework is usually referred to as Simultaneous Localization and Mapping (SLAM) [27]. Pose graph optimization has recently emerged as an effective solution for SLAM. This solution models the problem as an optimization problem over a factor graph where nodes represent poses assumed by a mobile robot at a certain time and edges represent sensor measurements [28].

Given the map of the surrounding environment, the robot needs also to be able to plan a path to reach a given point (global path planning) and to follow this path avoiding obstacles, like clutter and occupied areas, as well as people and other robots (local path planning). Classical algorithms performing global path planning are A${}^{★}$ and the more recent Rapidly exploring Random Tree (RRT${}^{★}$) [29].The most widely-used algorithms performing local panning are Vector Field Histograms (VFH+) [30] and the Dynamic Window Approach (DWA) [31].

Cloud robotics is a new trend in robotics. It leverages Internet-based technologies in order to allow an artificial agent to take advantage of the network resources to off-load computationally-intensive tasks. This naturally leads to a paradigm shift in which robots become simple agents that belong to a common cloud computing platform [32] and represents a further step in the direction of the Internet of Things [33,34]. In [35], the authors present Rapyuta, an open source Platform as a Service (PaaS) framework for robotics applications. Rapyuta is the engine underlying RoboEarth, a cloud robotics infrastructure, which aims at creating a World-Wide-Web-style database for storing knowledge generated by humans and robots in a machine-readable format. The potential of a cloud infrastructure opens a new world of possibility for service robotics [32,36,37,38,39,40], but introduces new research challenges [40].

Cloud robotics applications are becoming more and more popular thanks to the development of the Robot Operating System (ROS). ROS [41] is an open-source, meta-operating system for robotic software development. It provides a collection of packages and tools for the development of distributed robotic application. ROS is nowadays the de facto standard for robotic software development. The building blocks of ROS are the so-called nodes. A node is running a process in a ROS environment. Nodes interact with each other resorting on topics and services, which are, respectively, the ROS implementations of publish/subscribe and client/server communication patterns. The ROS framework simplifies the development of modular and distributed applications and suites well the cloud robotics paradigm.

## 2. The Proposed Solution

The proposed solution consists of a robotic platform equipped with environmental sensors, which are a thermal camera and a temperature/humidity sensor. The robot is completely autonomous, and after a simple configuration procedure, it is able to safety navigate in the data center environment.

The developed application is based on ROS and takes advantages of a cloud infrastructure that abstracts the underlying services and exposes them as RESTful APIs [42]. It also monitors the state of ROS nodes.

### 2.1. The Cloud Architecture

The proposed application is based on a cloud robotics platform developed by TIM S.p.A. The platform is based on ROS and on the concepts of Platform-as-a-Service (PaaS), presented in Rapyuta [43], and RObotics in CONcert(ROCON) [44]. The cloud robotics platform was first proposed in [2] and is able to abstract the hardware and software layers. It is able to offload demanding computational tasks and exposes simple RESTful APIs to the final user.

**Figure 2:**
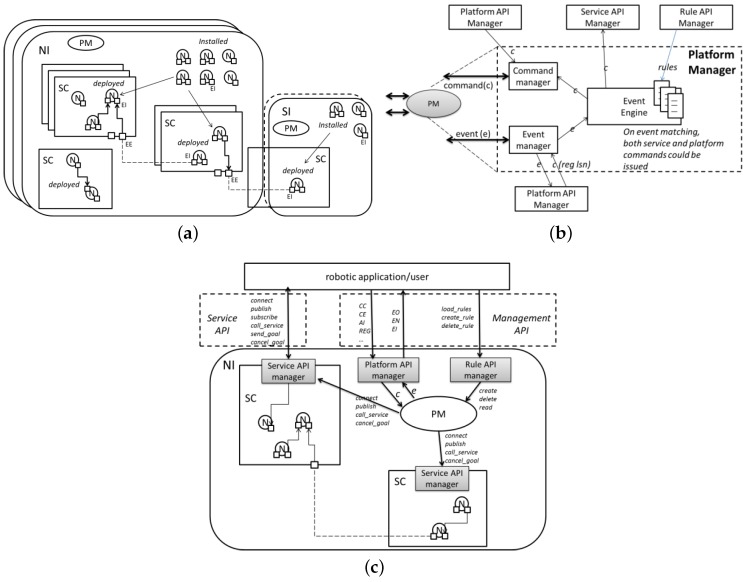
The cloud robotics platform developed by TIM. (**a**) The platform objects and their relationships; (**b**) the platform manager logic architecture; (**c**) the APIs.

The cloud robotics platform guarantees robustness in long-term applications: it stores the state of every ROS node in the application and is able to restart nodes that crash. The platform is also devoted to distribute the computational load among remote locations, providing better computational performances than the robot’s onboard PC. The basic elements of the cloud platform are depicted in Figure 2a and listed below.

- A Node (N) is a standard ROS node. It can be installed if it resides in an instance or started if it resides in a service container. It is connected if it has internal or external endpoints.
- A Service Container (SC) groups a set of nodes into a service.
- An Internal Endpoint (IE) connects nodes in the current service container or connects a node to an external endpoint.
- An External Endpoint (EE) connects nodes belonging to different service containers.
- The Instance is the object where the Platform Manager (PM) and the elements described before reside. The instance can be: Normal (NI) when it contains an SC and installed or started nodes, or Simple (SI) when it does not contain any SC, but only installed nodes.

These objects are used as building blocks to develop a distributed robotic application. The platform also provides RESTFul APIs to retrieve and change the application status. This is enabled by the Platform Manager (PM), depicted in Figure 2b. The PM can send and receive commands through the command manager. It can also listen to and create events through the event manager. Events and commands are accessed through the platform API manager. The event engine has a set of controlled counteractions triggered when previous configured classes of events occur. This has been conceived of to make the platform service robust and resilient. The counteractions can be both service commands (e.g., publish a message) and platform commands (e.g., create a service container).

The service API manager is a special node that needs to be started in the service container. It exposes APIs to the external world for managing service commands and events. External API managers, such as robotic applications and the event manager, access different kinds of APIs (Figure 2c): the platform API manager exposes APIs to manage the platform commands and events; the rule API manager exposes APIs to manage the procedures for the event engine.

### 2.2. The Cloud Architecture

**Figure 3:**
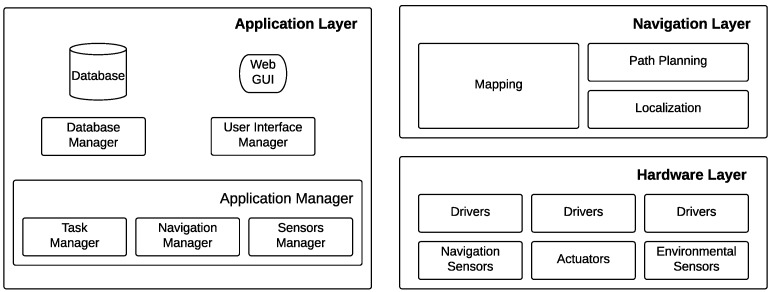
Functional architecture of the proposed solution.

The functional architecture (Figure 3) runs in a service container of the cloud robotics platform. It controls a single robot located in a server room in order to accomplish the monitoring task. It is composed of three layers:

- The hardware layer (Section 5.1) groups sensors and actuators (together with drivers) of the robotics platform: navigation sensors (e.g., laser range scanner, wheel encoders) are needed by navigation; environmental sensors (e.g., temperature/humidity probes, thermal camera) are required by the monitoring task.
- The navigation layer (Section 3) provides all of the capabilities related to autonomous navigation; it controls the robot in order to reach specific places within the environment by performing obstacle avoidance and adaptive path planning in order to deal with dynamic environments.
- The application layer (Section 4) controls the system in order to accomplish the given task.

## 3. The Navigation Layer

According to Section 2.2, the navigation layer groups all of the capabilities that allow a completely autonomous robot motion within the environment. The main capabilities are mapping, localization and path planning (Section 3.1).

The navigation layer works as a middleware between the hardware layer (that is the robot itself) and the application layer. It receives goal targets and controls the robot to reach each goal autonomously and safely.

### 3.1. Mapping, Localization and Path Planning

To solve the navigation problems, we take advantage of the ROS community and several open source packages available online.

The mapping procedures resorts on the gmapping ROS package [45]. gmapping is a state of the art solution to perform mapping that relies on Rao–Blackwellized particle filters [46]. During mapping, the robot is teleoperated from a simple web application. When the mapping procedure ends, the map is stored in the application database (see Section 4.3).

Robot localization resorts on the well-known Adaptive Monte Carlo localization (AMCL) algorithm first proposed in [47]. Monte Carlo localization approaches recursively estimate the posterior about the robot’s pose using particle filters.

The path planning module resorts on the move_base ROS package [48]. The package implements a two-stage path-planning procedure, composed by a global planner and a local planner based on state of the art solutions. Given the current robot pose, the map and a goal, the global planner finds a suitable path to reach the goal. The computed path is then executed by the local planner, which controls the robot in order to follow the path while performing obstacle avoidance. Local obstacles are detected using the laser scanner. Then, at each step, a number of local trajectories are simulated, and the best trajectory is chosen. At this point, the local planner converts the trajectory into velocity commands.

## 4. Application Layer

The application layer is devoted to the monitoring task. It is composed by three blocks: the application manager, the user interface manager and the database manager. The application manager controls the robot from a high-level point of view over the navigation capabilities, in order to allow the robot to reach a goal and collect data. The GUI manager exposes the web user interface in order to allow the user to interact with the system. The database manager manages the application data storage.

### 4.1. Database Manager

**Figure 4:**
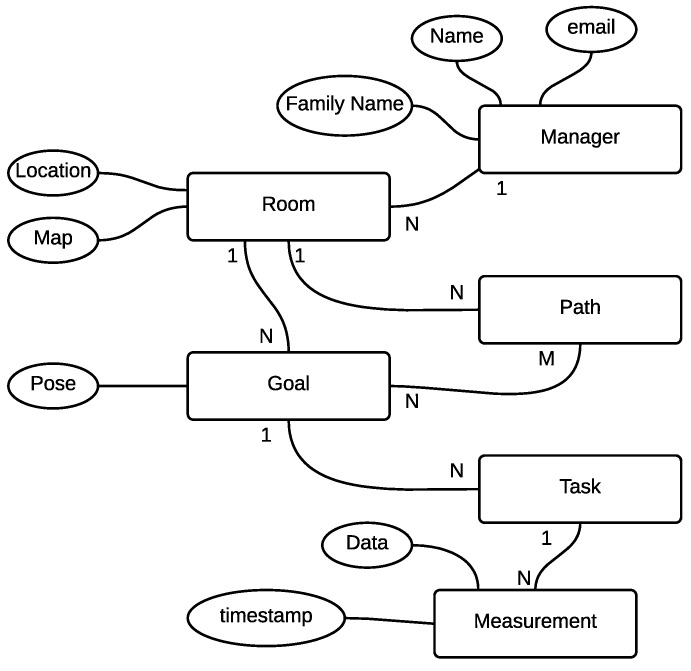
The database structure used in the proposed system. Rectangles represent tables, and ellipses represent data associated with each table. 1:N and M:N represent respectively one-to-many and many-to-many relationships.

The data collected by the service are stored in a database structured as depicted in Figure 4. The database is composed of four main tables, namely room, goal, task and plan. The room table models a server room. It groups general information (e.g., room manager information) and application-specific information, which are the map, a list of goal entries and a list of plan entries. The goal table models a 2D pose with an associated a set of task entries. The plan table is a list of goals within the same map. This table allows the service to perform different measurement campaigns in the same room. The task table represents tasks that the application has to perform when the corresponding goal is reached. Several actions have been implemented, such as collect data from the temperature/humidity sensor or collect data from the thermal camera. The docking action, when executed, starts the auto-docking procedure (see Section 5.4.4).

We call the Virtual Sensor Network (VSN) the set of goals associated with at least one measurement task. Each measurement task is a source of localized environmental measurements, i.e., a VSN node. A list of measurements entries storing the history of measurements is associated with each VSN node.

### 4.2. The Application Manager

The application manager is composed by the navigation manager, the task manager and the sensor manager.

The navigation manager is built on top of the navigation layer. It is devoted to sending high level goal positions to the navigation layer and managing the task to perform when a goal is reached. The manager sends robot goal positions to the navigation layer. When the application starts, the first plan is loaded in memory by the manager, and all goals belonging to the plan are sent one by one to the navigation layer. When all goals in the actual plan have been executed, the docking goal will be executed. After that, the process is repeated.

When the current goal is reached, the task manager is in charge of executing, one by one, all of the tasks associated with the current goal. Data acquisition tasks are managed by the sensor manager, which collects the data from sensors and stores them in the database. The docking task is managed by the navigation manager.

### 4.3. User Interface Manager

**Figure 5:**
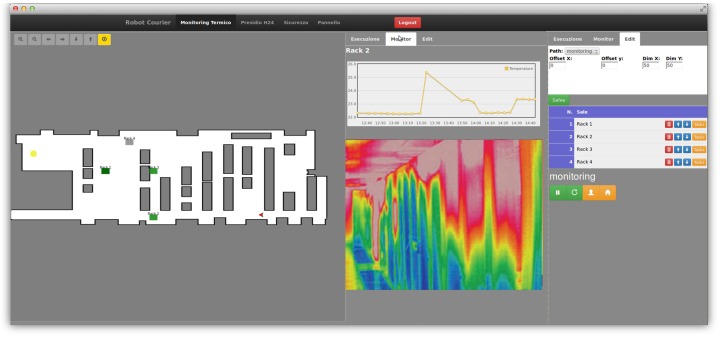
The web GUI.

We developed a web-based GUI (Figure 5), which provides an intuitive tool to access the data collected by the robot, as well as to monitor the state of the robot and control its motion. The GUI is connected with the underlying ROS nodes via RESTful APIs, which are exposed by the cloud infrastructure described in [1]. The web server exposing the GUI has been developed using the Flask Microframework [49], while the client has been developed over the Robot Web Tools [50].

Within the GUI, the user has full remote access to the robot capabilities: after a login form required for security, the user can monitor the robot state and position in the environment, access the acquired data stored in the database and send commands to the robot from both a high-level and low-level point of view. The user can send a goal to the navigation manager, pause the robot monitoring activity add or remove goal entries and edit path entries. It can also teleoperate the robot itself.

## 5. Experiments and Results

This section presents results coming from several experiments conducted to test both robot navigation performances and the entire application in real case scenarios. Our tests have been conducted in two data centers: the Rozzano 3 Data Center of TIM S.p.A. and the data center of Politecnico di Torino. In both cases, we installed the cloud robotic platform on a server placed in the same room in which the robot was located. In order to improve security, the robot platform and the cloud platform were connected over a dedicated WiFi connection setup inside the room, while remote access to the platform was enabled using a dedicated VPN.

Rozzano 3 is a large TIM S.p.A data center used for housing/hosting and internal services. It is located near Milan, Italy. It is composed of about twenty large server rooms (average area of 700 m${}^{2}$ per room). We had access to two rooms of the data center (Room 1 and Room 2 from now on). We tested the navigation part and the application for about one month of continuous operation. The data collected by the robot cannot be published due to a non-disclosure agreement with TIM S.p.A.

The data center of Politecnico di Torino is a small data center storing the University IT Infrastructure composed of three rooms of a small size (average area of 100 m${}^{2}$ per room). We had access to one room and tested the application in a real case scenario. This room is organized in three aisles with two rows of five racks, in order to have one isolated hot aisle and two cold aisles. The data center relies on an inter-racks cooling technology, i.e., each couple of adjacent racks is separated by a cooling module. The data center PUE has been computed as: $PUE=\frac{38.000\;\text{kW}}{24.710\;\text{kW}}=1.5378$.

### 5.1. Hardware Layer

**Figure 6:**
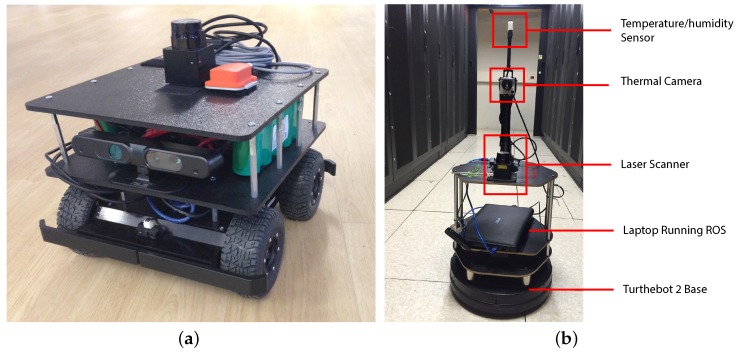
The two hardware platform prototypes. (**a**) The Coware Corob Classic 4 WD platform; (**b**) the Turtlebot 2 platform.

To demonstrate the usefulness of the cloud robot platform in abstracting the hardware layer and to be compatible with different robot platforms, we first tested our system on hardware from two different vendors: a Coroware Corobot Classic 4WD rover endowed with a Hokuyo 04LX laser range finder, an XSens MTi Inertial Measurement Unit (IMU) (Figure 6a) and a Turtlebot 2 robot platform endowed with a Hokuyo 04LX laser range finder (Figure 6b). Please note that the Turtlebot 2 platform is internally equipped with an IMU sensor. Both platforms were equipped with a PC running Ubuntu Linux and ROS.

We tested the navigation performances of both platforms in the Rozzano Data Center (see Section 5.4). The Turtlebot 2 robot was finally selected for the final prototype due to its robustness, integration with ROS and the presence of a built-in docking system.

The Turtlebot 2 robot platform was finally equipped with the environmental sensors: a thermal camera Optris PI 230 and a temperature/humidity sensor DHT22 controlled by an Arduino Uno board communicating with ROS using rosserial [51]. The thermal camera and the temperature/humidity sensor were mounted over a custom structure prototyped using fast prototyping solutions. The thermal camera has been positioned at a height of 0.8 m, while the temperature/humidity sensor at a height of 1 m.

### 5.2. Tuning Navigation Algorithms

The choice of the AMCL and move_base parameters is critical for achieving good performances in a challenging scenario. Good localization is crucial for the subsequent path planning, while correct planning greatly depends on the surrounding environment.

The most important parameter values that we use in our modified ROS implementation of the AMCL algorithm are reported in Table 1a. The error in laser readings caused by the metal grids (see Section 5.3) has been modeled in a trivial way by raising the laser_z_hit value of the likelihood field sensor model. The higher laser_z_rand value accounts for the presence of glass-covered racks. We experimentally found that a maximum particle size of 10,000 is enough for reliable global localization, and a minimum of 500 is enough for estimating the robot pose during position tracking.

Regarding path planning and path following, a tradeoff is needed between the ability of the robot to travel in narrow areas (such as narrow corridors between the racks) and the use of a safer distance from the obstacles in order to avoid possible collisions. We found in our experiments that the resolution of the rolling window has to be set higher than the default, and the inflation radius has to be set near the minimum possible value in order to ensure the robot will pass also in narrower corridors. The move_base package is also heavy on the computational side, so a tradeoff has to be made between accuracy and CPU load if the package is run onboard the robot. The values for the most important parameters that we use are shown in Table 1b.

### 5.3. Laser Issues

Data center spaces introduce specific problems that must be tackled in order to assure safe and reliable laser-based autonomous navigation. Metal grids covering racks introduce noise into laser scanner measurements. The noise is due to the fact that laser beams sometimes go through the holes of the grid. This phenomenon introduces errors in mapping and localization (see Section 5.4).

**Figure 7:**
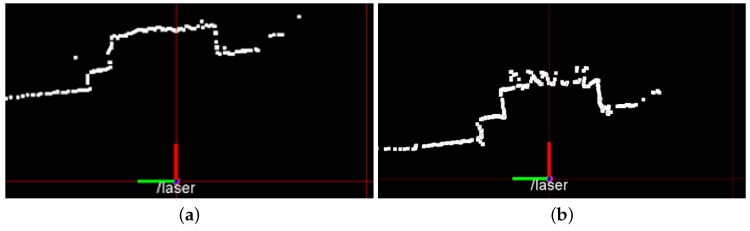
Noise caused by the grids on the laser range readings. Due to better angular resolution, the Hokuyo is affected by measurement noise due to metal grids covering the racks. (**a**) SICK LMS-200 laser scan; (**b**) Hokuyo 04LX laser scan.

To understand the entity of this issue, we test in the same position a SICK LMS-200 laser scanner (see Figure 7a) and the Hokuyo 04LX laser scanner (see Figure 7b) mounted on our robots. We found out that the SICK laser scanner was able to give more accurate distance measures, while the Hokuyo 04LX presents a significantly larger error in correspondence of the metal grids.

### 5.4. Robot Navigation

Here, we demonstrate the ability of the developed system to perform long-term navigation in the data center environment without issues. We conducted the navigation test campaign in Room 1 and Room 2 of the Rozzano data center, which were more challenging due to their dimensions. Please note that all of the navigation algorithms have been fully tested in different environments in the literature, so the aim of this section is to test navigation performances in the particular environment of data center rooms.

After a first set of experiments that were used to tune the parameters of the algorithms in order to maximize performances (see Section 5.2), we tested mapping, localization, path planning, path following, obstacles avoidance and docking.

#### 5.4.1. Experiment 1: Mapping

**Figure 8:**
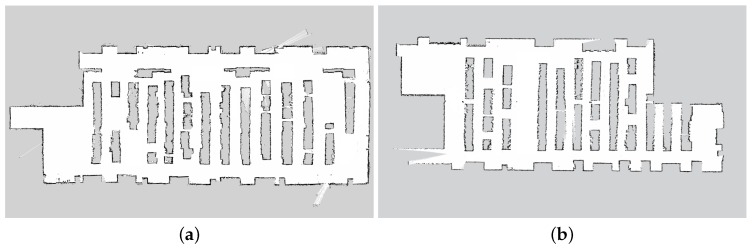
The two maps created with the mapping procedure. Due to the non-disclosure agreement, the map shown in these pictures has been partially edited in order to not reveal the real planimetry of the room. (**a**) Room 1; (**b**) Room 2.

The mapping step was executed in all of the data center rooms where we used the robot. In Figure 8, we report the maps created in Room 1 and Room 2 of the Rozzano data center. Both maps were created in approximately 30 min by manually tele-operating the robot. The resolution of the map is 0.05 m/pixel. The effect of metal grids can be seen, as the surfaces of the racks are irregular in some areas. Note that, due to the non-disclosure agreement with TIM, the maps shown in Figure 8 have been partially edited in order not to reveal the real planimetry of the rooms.

#### 5.4.2. Experiment 2: Localization, Path Planning and Path Following

**Figure 9:**
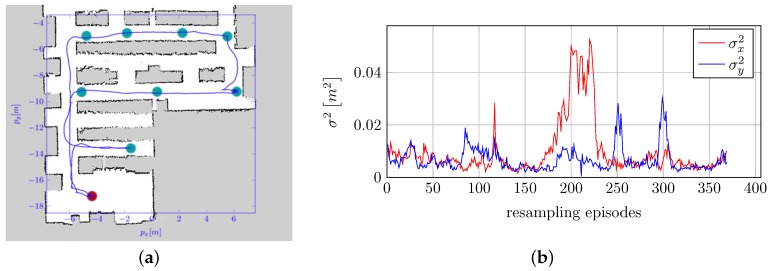
Localization and path planning performances. (**a**) Trajectory followed by the robot, as estimated by localization, is shown in blue; (**b**) estimated position error plot.

We evaluated the performances of localization, path planning and path following. A path composed of a certain number of goals was created within the environment, and the robot was controlled in order to reach each goal according to their order and then to re-execute the path. Figure 9a shows the results of a typical experiment. It can be noticed that the robot correctly localized itself and was able to follow the given path. Position localization errors are plotted in Figure 9b.

#### 5.4.3. Experiment 3: Obstacle Avoidance

We tested obstacle avoidance by performing Experiment 2 again in the presence of fixed and moving obstacles. We checked that the system was 100% able to avoid obstacles and re-planning the path if the current path is completely obstructed by obstacles.

#### 5.4.4. Experiment 4: Docking

We tested the performance of the docking system of the Turtlebot 2 robot platform. We set a navigation goal near the docking station and then controlled the robot in order to reach the goal using the navigation layer and then start the docking procedures. We executed the docking procedure 50 times, by making the robot start from a random initial position within the environment. The docking procedures failed three times and succeeded 47 times (94% success rate). Failures were caused by a non-perfect mechanical alignment between the robot and the docking station.

We improved the algorithm in order to re-execute the entire procedure if that docking station is not reached within a given time (e.g., 60 s). After that, we reached a 100% success rate.

#### 5.4.5. Experiment 5: Long-Term Navigation

We finally tested the whole system running on the Turtlebot 2 robot platform within Room 1 of the Rozzano data center. This final experiment was conducted during normal daily operations, with workers moving along the corridors, as well as obstacles, which were not present in the original map (open rack doors, carts, etc.). During this test, the robot was equipped with environmental sensors and was able to collect environmental data; however, due to the non-disclosure agreement, we are not allowed to publish the data collected.

We set a path of 80 goals and installed the docking station within Room 1 of the Rozzano Data Center. The system was programmed to execute the path 12 times a day (every 2 h) and then return to the docking station. Each mission lasted approximately 40 min. The experiment was run continuously for one month without human intervention. The only incident encountered was due to a cart, which was left for about one hour near the docking station. This cart prevented the robot from reaching the station and made it continuously execute the docking procedure until the cart was removed.

#### 5.4.6. Discussion

These experiments demonstrate that the developed system is able to correctly navigate within a data center environment. The errors introduced by the metal racks grids (see Section 5.3) propagate over the reconstruction of the maps, which present some irregular areas within aisles (Figure 8, Section 5.4.1) and over localization errors (Figure 9, Section 5.4.2), which are higher than the ones reported in other industrial applications [52]. However, localization is still adequate for the subsequent path planning.

Obstacle avoidance (Section 5.4.3), docking (Section 5.4.4) and long-term navigation (Section 5.4.4) do not present critical issues. Experiments guarantee the correct working of the system in long-term execution.

### 5.5. Monitoring Service

The entire monitoring service was tested in both the data center of TIM and that of Politecnico di Torino. These final tests aimed at demonstrating that the developed system is able to collect environmental data and to highlight some environmental management issues that the monitoring system of the data center was not able to point out. Despite the big difference in dimensions between the two data centers, both are similar in terms of layout and management. The small size does not affect the completeness of the experiments in the data center of Politecnico di Torino, since the ability of the robot to navigate in big environments has been already validated in Section 5.4.5.

In the Rozzano Data Center, the monitoring service was tested at the same time of Experiment 5 previously presented. As already said, the non-disclosure agreement prevents us from publishing the data collected during this campaign. However, we can report that we found some issues in energy management and layout of the data center, such as some hot spots.

Within the data center of Politecnico di Torino, we performed two intense data collection campaigns: the first one, performed 2 May 2016, lasted 14-h; the second one, performed between 25 and 26 May 2016, lasted 28 h. In order to allow the robot to monitor the whole environment, the isolation between the hot aisle and the rest of the room has been temporarily removed.

**Figure 10:**
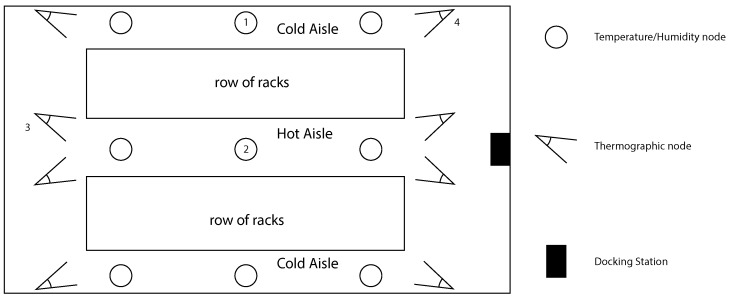
The data center layout and VSN nodes’ set-up. Triangles represent thermographic nodes, oriented in order to capture a thermographic image of half of the rack line’s side; circles represent temperature/humidity nodes, and rectangle represents the docking station.

We set the monitoring system as depicted in Figure 10 according to the data center manager instructions. We set three virtual temperature/humidity nodes per aisle (i.e., twelve nodes) and two thermographic VSN nodes per rack line’s side (i.e., eight nodes). The application was run in order to make the robot start a new test campaign after 10 min of recharging. The complete path is executed in about 6 min, resulting in capturing data in each VSN node with a sampling time of about 16 min.

#### 5.5.1. Collected Data

**Figure 11:**
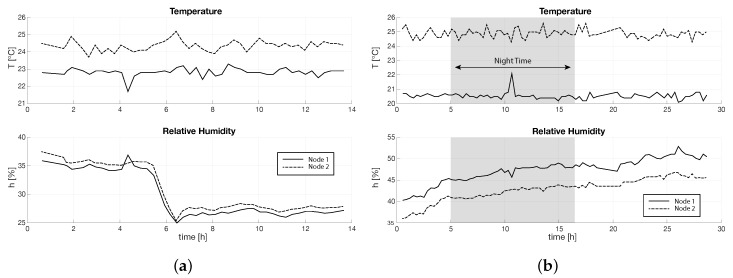
Temperature and relative humidity plots of data collected in the center VSN node in the hot aisle and in the center VSN node in one of the cold aisles. The grey region of (**b**) is the night period. (**a**) First campaign; (**b**) second campaign.

In Figure 11, we reported the temperature and humidity plots in time of two temperature/humidity VPN nodes, the former (Node 1) in the center of the the top aisle, the latter (Node 2) in the center of the hot aisle, as depicted in Figure 10. The plots of the other nodes are very similar. While the first campaign was done during the day time, the second campaign ran also during night time. We highlighted in Figure 11b the night period, from 8 p.m. of the first day to 8 a.m. of the second day.

**Figure 12:**
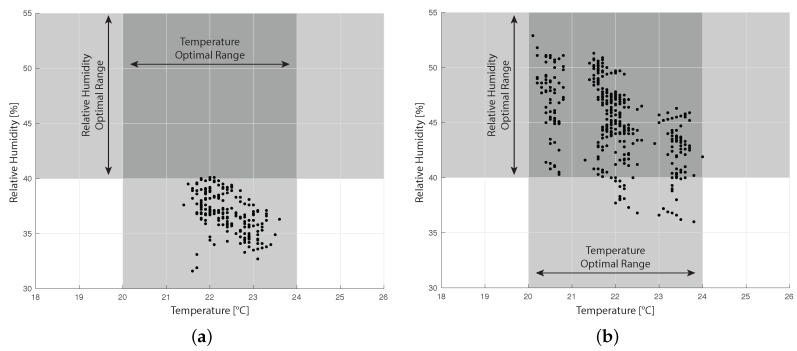
Relative humidity vs. temperature plots of cold air data. The grey zone represents the optimal suggested values of temperature and humidity to maximize the hardware life cycle. (**a**) First campaign; (**b**) second campaign.

Figure 12 reports the relative humidity vs. temperature plots of the cold aisles collected in both experiments. We also reported in grey the optimal ranges of temperature and humidity that maximize the hardware life cycle, according to [10]. Note that optimal ranges are related to air that is used to cool the system; hence, only the data collected in the cold aisles are reported.

**Figure 13:**
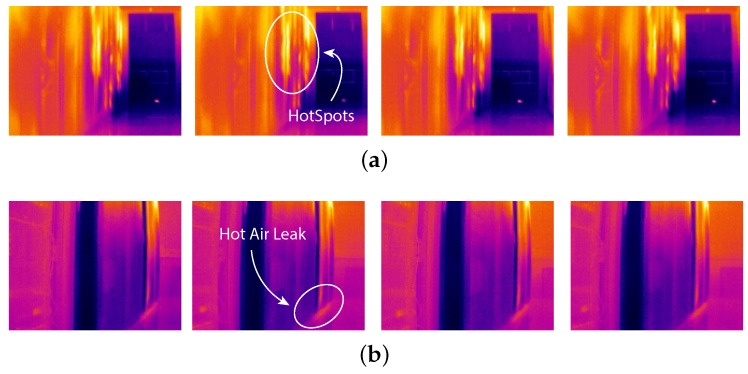
Thermographic analysis of two thermographic nodes at different times. Images have been captured in the second data collection at times 1 h, 10 h, 20 h and 27 h. (**a**) Thermographic Node 3; (**b**) Thermographic Node 4.

Figure 13 reports thermographic images captured at different times during the second campaign (i.e., 1 h, 10 h, 20 h and 27 h) from Node 3 and Node 4 (see Figure 10).

#### 5.5.2. Discussion

Collected data reported in Figure 12 show that the humidity in the room is not well maintained. During the first campaign, we measured a huge decrease in relative humidity that went under 30% (see Figure 12a). In the second campaign, the humidity slowly increased (see Figure 12b). We compared these data to the external humidity data given by the weather history, and we noticed a similar trend. This may point to issues in the isolation of the data center room. On the other hand, temperature seems to be well controlled in the environment: in fact, in both campaigns, we can see a stable trend in time. However, data show a difference in temperature between the two campaigns. Node 1 presents an average temperature of 22.8 C during the first campaign and of 20.6 C during the second campaign. On the other hand, Node 2 average temperature increases from 24.4 C to 24.9 C This phenomenon is probably due to a higher external temperature that requires a higher cooling capacity of the air: average external temperature was 13 C during the first campaign and 17 C during the second campaign. This confirms the fact that the data center is not well isolated from the external environment.

The reliability of the temperature control system is also verified by Figure 12, which compares collected data about cold air with the optimal ranges that maximize the hardware life cycle [10]. Figure 12 points out that the temperature of cold air is always within the optimal range. On the other hand, relative humidity values are strongly out of range in the first campaign (Figure 12a) and partially within the optimal range in the second campaign (Figure 12b). This fact highlights the necessity to improve the humidity control system.

Figure 13a shows the presence of some hotspots in the hot aisle; however, this is not a big issue since, usually, the hot aisle is isolated from the rest of the environment. On the other hand, in Figure 13b, we can notice a small hot air leak from the bottom of a rack. This leak is an issue in thermal efficiency, since it causes hot air to recycle and, hence, an over-cooling phenomenon near the rack. We found out, with the help of the data center manager, that the leak was caused by a network switcher not correctly placed within the rack.

Finally, Figure 12b points out that there is no difference in terms of thermal load between day and night. Since the data center is a university data center, we expected the computational load of the data center to strongly decrease during the night. However, the data center is managed in order to perform high computation background procedures during the night, such as backups, that cause an average computational load that is very similar during day and night.

## 6. Conclusions

In this paper, we presented a service robotics application for solving the problem of environmental monitoring of data centers. Our solution relies on a cloud robotics framework based on the Robot Operating System (ROS) and simulates an ESN. Instead of using several fixed sensors (nodes), a few sensors mounted on an autonomous mobile base are sufficient to reach each point of the environment and give localized measures. We called this system a Virtual Sensor Network (VSN).

We described the general architecture of the cloud robotics platform and how we applied this technology in a real service robotics application. Then, we described the application itself and our choices related to robot navigation and the monitoring application.

Finally, we showed the results from several experiments in order to validate the ability of the proposed solution to autonomously navigate within the environment (using two different robotic hardware platforms) and to test the application in real case scenarios. Data collection and thermographic analysis performed inside the data center room of Politecnico di Torino highlighted some issues regarding humidity control and isolation, which could be reported to the data center management for action.

## Figures and Tables

**Table 1:** The parameters used for the AMCL and mobe_base ROS packages. (**a**) Parameters used for AMCL ROS node; (**b**) parameters used for move_base node.

Parameter	Value
(**a**)	
max_particles	10,000
min_particles	500
laser_z_hit	0.5
laser_z_rand	0.5
update_min_d	0.1
update_min_a	0.25
resample_interval	1
(**b**)	
resolution	0.05
inflation_radius	0.35
transform_tolerance	3
path_distance_bias	1.0

## Supplementary Materials

A video of the proposed system while working is available online at https://www.youtube.com/watch?v=HlUB0oHuXrc.

## Abbreviations

The following abbreviations are used in this manuscript:

AGV: Automated Guided Vehicles
(A)MCL: (Adaptive) Monte Carlo Localization
API: Dynamic Window Approach
EKF: Extended Kalman Filters
ESN: Environmental Sensor Network
GUI: Graphical User Interface
IMU: Inertial Measurement Unit
NFC: Near Field Communication
PaaS: Platform as a Service
PM: Platform Manager
PUE: Power Usage Effectiveness
REST: REpresentational State Transfer
ROCON: RObotics in CONcert
ROS: Robot Operating System
RRT${}^{★}$: Rapidly exploring Random Tree
SLAM: Simultaneous Localization and Mapping
VFH+: Vector Field Histograms
VSN: Virtual Sensor Network

## References

[1] Rosa S., Russo L.O., Airó Farulla G., Carlone L., Antonini R., Marco G., Bona B. An application of laser-based autonomous navigation for data-center monitoring. Proceedings of the 13th International Conference IAS-13.

[2] Rosa S., Russo L.O., Bona B. Towards a ROS-based autonomous cloud robotics platform for data center monitoring. Proceedings of the 2014 IEEE Emerging Technology and Factory Automation (ETFA).

[3] The 2020 Climate and Energy Package, EU Climate Action. http://ec.europa.eu/clima/policies/strategies/2020/indexen.htm.

[4] Koomey J. (2011). Growth in Data Center Electricity Use 2005 to 2010.

[5] Patterson M.K. The effect of data center temperature on energy efficiency. Proceedings of the 11th Intersociety Conference on Thermal and Thermomechanical Phenomena in Electronic Systems.

[6] Brill K.G. (2007). Data Center Energy Efficiency and Productivity.

[7] Anandan S.S., Ramalingam V. (2008). Thermal management of electronics: A review of literature. Therm. Sci..

[8] Capozzoli A., Primiceri G. (2015). Cooling systems in data centers: State of art and emerging technologies. Energy Procedia.

[9] Villars R.L. (2012). The Datacenter’s Role in Delivering Business Innovation.

[10] Wang L., Khan S.U. (2013). Review of performance metrics for green data centers: A taxonomy study. J. Supercomput..

[11] Patel C.D., Bash C.E., Belady C., Stahl L., Sullivan D. Computational fluid dynamics modeling of high compute density data centers to assure system inlet air specifications. Proceedings of the Pacific Rim/ASME International Electronic Packaging Technical Conference and Exhibition.

[12] Patel C.D., Bash C.E., Sharma R., Beitelmal M., Friedrich R. Smart cooling of data centers. Proceedings of the ASME 2003 International Electronic Packaging Technical Conference and Exhibition.

[13] Ranganathan P., Leech P., Irwin D., Chase J. Ensemble-level Power Management for Dense Blade Servers. ACM SIGARCH Computer Architecture News. http://www.ecs.umass.edu/~irwin/hp.pdf.

[14] Bash C.E., Patel C.D., Sharma R.K. Dynamic thermal management of air cooled data centers. Proceedings of the Tenth Intersociety Conference on Thermal and Thermomechanical Phenomena in Electronics Systems (ITHERM’06).

[15] Nathuji R., Isci C., Gorbatov E. Exploiting platform heterogeneity for power efficient data centers. Proceedings of the the Fourth International Conference on Autonomic Computing (ICAC’07).

[16] Das R., Kephart J.O., Lefurgy C., Tesauro G., Levine D.W., Chan H. Autonomic multi-agent management of power and performance in data centers. Proceedings of the 7th International Joint Conference on Autonomous Agents and Multiagent Systems: Industrial Track.

[17] Parolini L., Sinopoli B., Krogh B.H. Reducing data center energy consumption via coordinated cooling and load management. Proceedings of the Proceedings of the 2008 Conference on Power Aware Computing and Systems.

[18] Choi W., Park K.-W., Park K.H. Scout: Data center monitoring system with multiple mobile robots. Proceedings of the 2011 7th International Conference on Networked Computing and Advanced Information Management (NCM).

[19] Lenchner J., Isci C., Kephart J.O., Mansley C., Connell J., McIntosh S. Towards data center self-diagnosis using a mobile robot. Proceedings of the 8th ACM International Conference on Autonomic Computing, ICAC 2011.

[20] Kiva Systems. http://www.kivasystems.com.

[21] Bona B., Carlone L., Indri M., Rosa S. (2014). Supervision and monitoring of logistic spaces by a cooperative robot team: Methodologies, problems, and solutions. Intell. Serv. Robot..

[22] Guizzo E. (2008). Three engineers, hundreds of robots, one warehouse. IEEE Specturm.

[23] Hamann H.F., Schappert M., Iyengar M., van Kessel T., Claassen A. Methods and techniques for measuring and improving data center best practices. Proceedings of the IEEE 11th Intersociety Conference on Thermal and Thermomechanical Phenomena in Electronic Systems, ITHERM 2008.

[24] Hamann H.F., van Kessel T.G., Iyengar M., Chung J.Y., Hirt W., Schappert M.A., Claassen A., Cook M.J., Min W., Amemiya Y. (2009). Uncovering energy-efficiency opportunities in data centers. IBM J. Res. Dev..

[25] Vis I.F.A. (2006). Survey of research in the design and control of automated guided vehicle systems. Eur. J. Oper. Res..

[26] Kelly A., Nagy B., Stager D., Unnikrishnan R. (2007). Field and service applications—An infrastructure-free automated guided vehicle based on computer vision—An effort to make an industrial robot vehicle that can operate without supporting infrastructure. IEEE Robot. Autom. Mag..

[27] Thrun S., Burgard W., Fox D. (2005). Probabilistic Robotics.

[28] Lu F., Milios E. (1997). Globally consistent range scan alignment for environment mapping. Auton. Robots.

[29] Lavalle S.M. Rapidly-Exploring Random Trees: A New Tool for path planning.

[30] Ulrich I., Borenstein J. Vfh+: Reliable obstacle avoidance for fast mobile robots. Proceedings of the 1998 IEEE International Conference on Robotics and Automation.

[31] Fox D., Burgard W., Thrun S., Fox D., Burgar W. (1997). The dynamic window approach to collision avoidance. IEEE Trans. Robot. Autom..

[32] Waibel M., Beetz M., Civera J., d’Andrea R., Elfring J., Galvez-Lopez D., Haussermann K., Janssen R., Montiel J.M.M., Perzylo A. (2011). A world wide web for robots. IEEE Robot. Autom. Mag..

[33] Iera A., Atzori L., Morabito G. (2010). The internet of things: A survey. Comput. Netw..

[34] Hagita N., Sanfeliu A., Saffiotti A. (2008). Network robot systems. Robot. Auton. Syst..

[35] Hunziker D., Gajamohan M., Waibel M., D’Andrea R. Rapyuta: The roboearth cloud engine. Proceedings of the 2013 IEEE International Conference on Robotics and Automation (ICRA).

[36] Du Z., Chen Y., García-Acosta M. Robot as a service in cloud computing. Proceedings of the 2010 Fifth IEEE International Symposium on Service Oriented System Engineering (SOSE).

[37] Menezes P., Quintas J., Dias J. Cloud robotics: Towards context aware robotic networks. Proceedings of the International Conference on Robotics.

[38] Hagita N., Kamei K., Nishio S., Sato M. (2012). Cloud networked robotics. IEEE Netw..

[39] Chibani A., Amirat Y., Mohammed S., Matson E., Hagita N., Barreto M. (2013). Ubiquitous robotics: Recent challenges and future trends. Robot. Auton. Syst..

[40] Kehoe B., Patil S., Abbeel P., Goldberg K. (2015). A survey of research on cloud robotics and automation. IEEE Trans. Autom. Sci. Eng..

[41] Quigley M., Conley K., Gerkey B., Faust J., Foote T., Leibs J., Wheeler R., Ng A.Y. (2009). ROS: An open-source robot operating system. ICRA Workshop Open Source Softw..

[42] Richardson L., Ruby S. (2008). RESTful Web Services.

[43] Mohanarajah G., Hunziker D., D’Andrea R., Waibel M. (2015). Rapyuta: A cloud robotics platform. IEEE Trans. Autom. Sci. Eng..

[44] Robotics in Concert. http://www.robotconcert.org.

[45] Gmapping ROS Package. http://wiki.ros.org/gmapping.

[46] Grisetti G., Stachniss C., Burgard W. (2007). Improved techniques for grid mapping with rao-blackwellized particle filters. IEEE Trans. Robot..

[47] Fox D. (2001). Kld-sampling: Adaptive particle filters. Advances in Neural Information Processing Systems 14.

[48] Move_Base ROS Package. http://wiki.ros.org/move_base.

[49] the Flask Microframework. http://flask.pocoo.org/.

[50] Robot Web Tools. http://robotwebtools.org/tools.html.

[51] The Rosserial Package. http://wiki.ros.org/rosserial.

[52] Rowekamper J., Sprunk C., Tipaldi G.D., Stachniss C., Pfaff P., Burgard W. On the position accuracy of mobile robot localization based on particle filters combined with scan matching. Proceedings of the 2012 IEEE/RSJ International Conference on Intelligent Robots and Systems (IROS).

